# Organ Preservation in Colon Cancer: An Illustrative Case Report

**DOI:** 10.7759/cureus.25351

**Published:** 2022-05-26

**Authors:** Kruti B Vora, Sameer Tolay, Aparna R Parikh, Sakti Chakrabarti

**Affiliations:** 1 Internal Medicine, Massachusetts General Hospital, Harvard Medical School, Boston, USA; 2 Oncology, SSM Health St. Agnes Hospital, Fond du Lac, USA; 3 Medicine, Massachusetts General Hospital, Harvard Medical School, Boston, USA; 4 Medical Oncology (GI Oncology), Medical College of Wisconsin, Milwaukee, USA

**Keywords:** immune checkpoint inhibitor, circulating tumor dna, immunotherapy, organ preservation, colon cancer

## Abstract

The organ preservation strategy in non-metastatic rectal cancer is a rapidly evolving, novel treatment paradigm that is offered outside of a clinical trial in many advanced cancer centers. However, for non-metastatic colon cancer, upfront surgery followed by adjuvant chemotherapy in patients deemed at risk of cancer recurrence is the current standard of care. A significant proportion of patients with non-metastatic colon cancer harbor a deficient mismatch repair (dMMR)/microsatellite instability-high (MSI-H) signature in tumors, which predicts a deep and durable response to immune checkpoint inhibitors (ICI) in a large proportion of such patients. This opens an opportunity for organ preservation in colon cancer in select circumstances. Herein, we describe a patient with locally advanced dMMR/MSI-H colon cancer who could not undergo standard colon surgery but achieved complete remission following treatment with ICI.

## Introduction

Rapid advances in the understanding of tumor genomics relevant to cancer growth and migration have led to the recognition of several therapeutically important distinct subsets of colorectal cancer (CRC). One well-recognized subset of CRC is tumors with deficient mismatch repair mechanism (dMMR), leading to high microsatellite instability (MSI-H), which is present in approximately 15% of non-metastatic CRC patients [1]. Mismatch repair deficiency in CRC could be secondary to hereditary mutations in mismatch repair (MMR) genes as in Lynch syndrome or sporadic epigenetic change causing promoter hypermethylation of MLH1 (accounting for 80% of dMMR CRC) [1]. As a result of mismatch repair impairment, cells fail to recognize and repair DNA damages, resulting in the accumulation of mutations that lead to neoantigen formation [2]. Neoantigens are recognizable by the immune system as foreign antigens that form the biological basis of the exquisite responsiveness of dMMR/MSI-H tumors to immune checkpoint inhibitors (ICIs) [2].

The current standard treatment of non-metastatic colon cancer is upfront surgery [3]. However, occasionally, systemic therapy is administered before surgery to shrink the tumor if the extent of the tumor makes a margin-negative resection unlikely [3]. Furthermore, patients with non-metastatic colon cancer are sometimes treated with systemic treatment alone if co-morbidities preclude surgery. A plethora of studies have recently reported deep and durable responses with ICIs in dMMR/MSI-H tumors [1,4-5]. Consequently, if surgery cannot be pursued in a patient with dMMR/MSI-H non-metastatic CRC for any reason, the administration of an ICI is an acceptable treatment choice, as illustrated by the current case report.

## Case presentation

**Figure 1:**
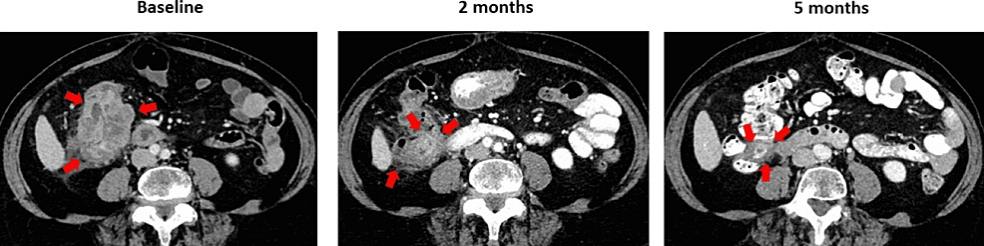
Serial CT scans at baseline, two months, and five months after immunotherapy initiation with ipilimumab and nivolumab combination demonstrating progressive shrinkage of the right hepatic flexure mass (indicated by red arrows).

A 75-year-old woman presented with progressive nausea, upper abdominal pain, abdominal bloating, and fatigue developing over three to four months. She also had a reduced appetite and about 20 pounds of weight loss. She reported passing soft stools several times a day but denied blood in her stool or hematemesis. A review of past medical history revealed hypertension and atrial fibrillation controlled with medications. Her complete blood counts and chemistry panel were unremarkable. A CT scan of the chest, abdomen, and pelvis revealed a large hepatic flexure mass encroaching onto the duodenum and head of the pancreas associated with regional lymphadenopathy but without complete bowel obstruction (Figure 1).

**Figure 2:**
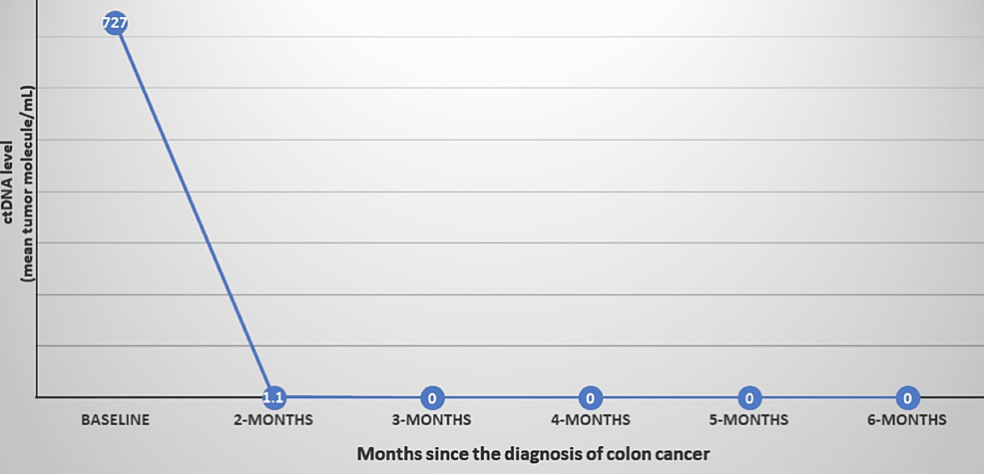
Serial circulating tumor DNA (ctDNA) levels during the treatment, with immunotherapy demonstrating a sharp drop and eventual clearance of ctDNA with immunotherapy treatment

The CT scan did not show any distant metastasis (clinical stage III colon cancer). A colonoscopy confirmed an intraluminal hepatic flexure mass. A colonoscopic biopsy from the mass confirmed a poorly differentiated adenocarcinoma. Immunohistochemistry (IHC) for MMR proteins revealed loss of MLH1 and PMS2, confirming a diagnosis of dMMR tumor. Tumor genomic profiling revealed BRAF V600E mutation suggesting sporadic colon cancer. Her carcinoembryonic antigen (CEA) level at diagnosis was 1.0 ng/mL (normal range ≤ 4.7 ng/mL). She was referred to a colorectal surgeon who expressed concern about a high likelihood of residual disease at the margin with upfront surgery. Furthermore, there was a concern that without neoadjuvant therapy, the patient would require a pancreaticoduodenectomy in addition to a right hemicolectomy. A discussion in the multidisciplinary colorectal tumor board confirmed the concerns. As a result, the patient was treated with ipilimumab 1 mg/Kg every six weeks and nivolumab 3 mg/Kg every two weeks (Ipi/Nivo) based on the NICHE (Nivolumab, Ipilimumab, and COX2-inhibition in Early Stage Colon Cancer: an Unbiased Approach for Signals of Sensitivity) study [6] and CheckMate 142 study [5] data (discussed in the following section), which she started about four weeks after the diagnosis. A CT scan after two months and five months following initiation of the treatment with ICI showed progressive shrinkage of the tumor (Figure 1). In addition, blood samples were obtained to measure circulating tumor DNA (ctDNA) levels using the commercially available Signatera^TM^ (Natera, Austin, TX) test [7] at baseline and serially, which showed a dramatic decrease followed by the disappearance of ctDNA (Figure 2).

The patient experienced remarkable symptomatic improvements with complete resolutions of all her symptoms, including abdominal pain, nausea, anorexia, and abdominal bloating in two months. She also gained weight with marked improvement in her strength. At this point, surgery was offered, which the patient did not want to pursue considering the possibility that she was in complete remission and possible complete pathological responses (pCR; discussed in the following section). Subsequently, a repeat colonoscopy performed six months after the initial colonoscopy showed mild narrowing of the hepatic flexure without residual cancer. Several biopsies obtained from the tumor site did not reveal any malignancy on histological examination. However, the patient developed immune-related hypophysitis, manifested by extreme fatigue, nausea, lightheadedness, low adrenocorticotropic hormone (ACTH), and cortisol four months after initiation of the ICI after receiving three doses of ipilimumab and seven doses of nivolumab, which responded well to oral steroid supplementation. ICI has been discontinued at this time. Currently, she is being followed with serial CT scans and ctDNA tests off treatment. Her last evaluation seven months after diagnosis did not show any evidence of cancer relapse.

## Discussion

The patient described in the current case report is a 75-year-old woman presenting with symptomatic locally advanced dMMR/MSI-H hepatic flexure adenocarcinoma, which was successfully managed with an ICI combination (Ipi/Nivo). Her most recent evaluation with a colonoscopy and ctDNA showed a complete response (CR). This case report illustrates a unique scenario in which a patient with locally advanced colon cancer has been managed without surgery, although upfront surgery is the current standard of care for such patients [3], providing a hint that organ preservation could be feasible in a select group of localized colon cancer patients harboring dMMR/MSI-H signature.

For many decades, upfront surgery followed by adjuvant chemotherapy in patients deemed at risk of cancer recurrence has been the standard of care for patients with non-metastatic colon cancer [3]. However, neoadjuvant therapy is endorsed by the expert panel in select circumstances, especially when the extent of the tumor makes margin-negative resection unlikely [3]. Consequently, pursuing systemic treatment with ICI rather than upfront surgery was logical for the patient under discussion. We chose to treat her with Ipi/Nivo combination based on the data presented in the NICHE trial [6]. However, we decided on an extended treatment duration with the regimen utilized in the CheckMate142 (A Study of Nivolumab Alone or Nivolumab Combination Therapy in Colon Cancer That Has Come Back or Has Spread) study [5], given the large size of the tumor and extensive local invasion.

The NICHE study is one of the earlier studies investigating the safety and feasibility of neoadjuvant immunotherapy with Ipi/Nivo in patients with early-stage colon cancer [6]. A total of 40 early-stage CRC patients with 21 dMMR and 20 proficient MMR (pMMR) tumors were enrolled. Patients received one dose of ipilimumab (1 mg/Kg) and two doses of nivolumab (3 mg/Kg) two weeks apart, and the surgery was performed within six weeks of enrollment. The primary objective was safety and feasibility. The treatment was found to be safe and well-tolerated, and all patients underwent planned curative resection without any treatment-related delay. The most notable finding of the study was pathological responses in 20 out of 20 dMMR tumors treated, with 19 major pathological responses (MPR) defined as the presence of ≤10% viable residual tumor and 12 complete pathological responses (pCR). All dMMR colon cancer patients were alive and disease-free at a median follow-up of 8.1 months. In patients with pMMR tumors, four out of 15 (27%) showed pathological responses, with three MPRs and one partial response. The correlative studies reported that CD8+PD-1+ T cell infiltration was predictive of response in pMMR tumors. The NICHE study was the first study exploring the utility of neoadjuvant immunotherapy in localized colon cancer and will likely pave the way for neoadjuvant immunotherapy as standard therapy for localized dMMR early-stage colon cancer. This study has been expanded to 60 patients and currently recruiting (NCT03026140; accessed on May 7, 2022).

We chose an extended treatment regimen as used in the CheckMate142 study [5], given the large size of the tumor and extensive local invasion by the tumor. In the CheckMate142 study, patients with metastatic dMMR/MSI-H CRC with no prior treatment were treated with nivolumab 3 mg/Kg once every two weeks and ipilimumab 1 mg/Kg once every six weeks until disease progression. The study reported an objective response rate of 69%, with a 13% complete response rate. Median progression-free and overall survival were not reached with a median follow-up of 29 months and a minimum follow-up of 24.2 months. The high probability of deep and durable response to ICI in dMMR/MSI-H patients supports utilizing immunotherapy in the neoadjuvant setting. Neoadjuvant immunotherapy might play a prominent role for dMMR/MSI-H localized CRC in the near future, as biomarkers that predict response to immunotherapy in dMMR/MSI-H tumors (for example, T cell density score [8]) are validated in larger studies.

We chose to obtain ctDNA levels for tumor response, monitoring for several reasons. First, radiologic studies often do not provide clear guidance regarding treatment response in dMMR/MSI-H patients [9]. Second, the INSPIRE (Probable Benefit of the Neuro-Spinal Scaffold for Treatment of AIS A Thoracic Acute Spinal Cord Injury) study [10] demonstrated long-term remission in dMMR/MSI-H solid-tumor patients achieving complete disappearance of ctDNA after immunotherapy, which provides a rationale for deferring surgery in patients with ctDNA turning negative after immunotherapy. Third, the serum CEA level in this patient was non-informative, as the level was within the normal range at the time of diagnosis. Finally, patients treated with non-standard neoadjuvant therapy need close monitoring for cancer relapse to enable the prompt reintroduction of treatment if cancer relapses. In this context, ctDNA has shown remarkable promise, as ctDNA can predict cancer relapse with a mean lead time of 8.7 months compared to radiologic studies [7]. Tumor response monitoring with serial ctDNA level measurements might evolve as a novel tool in the near future.

It is important to emphasize that 60% of patients achieved pCR after treatment with just one dose of ipilimumab and two doses of nivolumab in the NICHE study [6]. Furthermore, Ludford et al. reported pCR in 13 out of 14 resected patients of dMMR/MSI-H metastatic CRC who had residual tumors in the radiologic studies after preoperative treatment with immunotherapy [9]. Based on these data, it is reasonable to assume that the patient under discussion has already achieved pCR after receiving three doses of ipilimumab and seven doses of nivolumab, especially since the repeat colonoscopy did not show any residual colonic tumor. Consequently, we chose to observe her closely with serial ctDNA and CT scan rather than reintroducing immunotherapy. If she develops tumor recurrence, retreatment with ICI would be a consideration, as demonstrated in the GERCOR NIPICOL (Interest of iRECIST Evaluation for DCR for Evaluation of Patients With Deficient MMR and /or MSI Metastatic Colorectal Cancer Treated With Nivolumab and Ipilimumab) study [11].

It is important to emphasize that patients undergoing treatment with ICIs should be monitored closely for immune-related adverse events (IRAEs). Grade 3 or higher IRAEs can develop in 22% of patients treated with the Ipi/Nivo combination [5]. Furthermore, if the steroid is needed to control toxicities, the steroid should be administered without hesitation, as retrospective data suggest that steroid administration to control IRAEs does not affect survival outcomes [12].

## Conclusions

Non-metastatic colon cancer patients harboring the dMMR/MSI-H signature can be successfully treated with ICI if upfront surgery is not feasible. In a significant number of patients, ICI therapy can result in complete remission that may justify deferring surgery altogether in carefully selected patients. ctDNA-guided monitoring of tumor response is a promising evolving paradigm.
